# Supplementary material for: Repulsion leads to coupled dislocation motion and extended work hardening in bcc metals
Source: Nat Commun. 2020 Oct 9;11:5098. doi: 10.1038/s41467-020-18774-1 (PMC7547675; doi:10.1038/s41467-020-18774-1)
Supplement: Supplementary file 1 — Supplementary Information [file 41467_2020_18774_MOESM1_ESM.pdf]

Repulsion leads to coupled dislocation motion and extended work-hardening in bcc metals

Srivastava et al.

Supplementary Figures

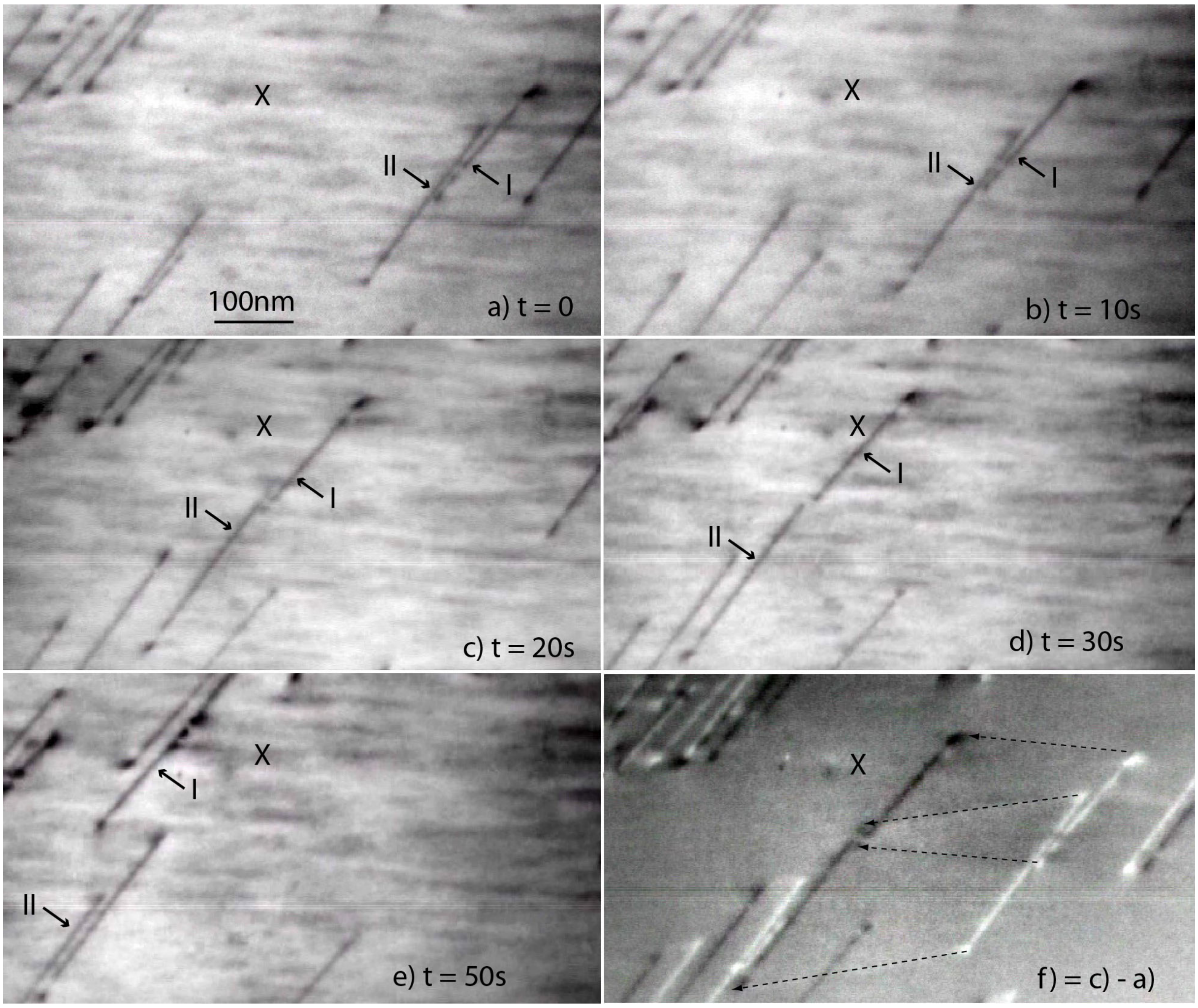

Supplementary Figure 1: Experimental Evidence of coupling in Fe

Same dislocation coupling as in fig. 3, in pure iron strained at 95K. Foil normal $[\bar{1}34]$, tensile axis $[\bar{7}3\bar{4}]$, diffraction vector $(\bar{2}\bar{1}1)$. Dislocations I and II have Burgers vectors $\mathbf{b}_{I}$ = 1/2$[\bar{1}11]$ and $\mathbf{b}_{II}$ = 1/2 [111], respectively. The point of closest distance moves along the $[\bar{1}\bar{1}1]$ direction which is at the intersection of the two glide planes, respectively (101) and $(\bar{1}10)$. Dislocation I pushes dislocation II between (a) and (c) (see difference-image (f)). Then the two dislocations are uncoupled (d), and dislocation I glides faster than dislocation II (d, e). X is a fixed point.

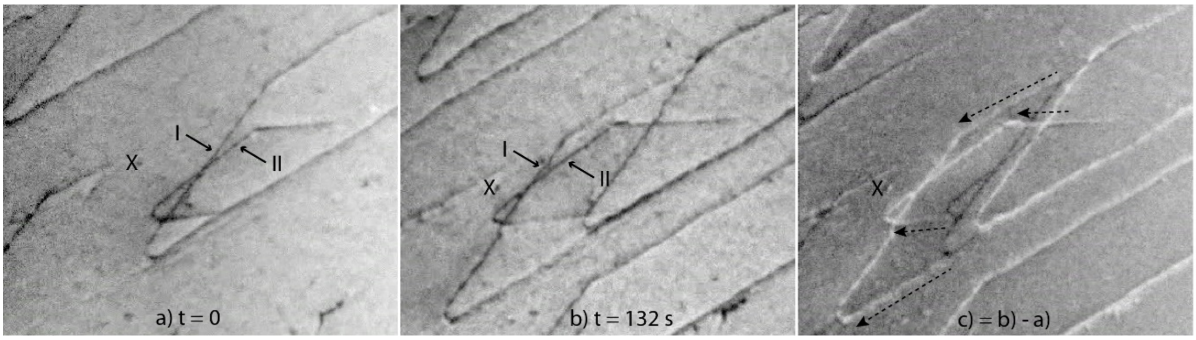

Supplementary Figure 2: **Experimental Evidence of coupling in Nb**: Same dislocation coupling as in fig. 3, in niobium strained at 95K. Foil normal $[8\bar{1}0]$, tensile axis $[\bar{1}\bar{8}0]$, diffraction vector $(01\bar{1})$. Dislocations I and II have Burgers vectors $\mathbf{b}_I$=1/2[111] and $\mathbf{b}_{II} = 1/2[1\bar{1}\bar{1}]$ respectively. The point of closest distance moves along the$[1\bar{1}\bar{3}]$ direction which is at the intersection of the two glide planes, respectively $(1\bar{2}1)$ and (110).

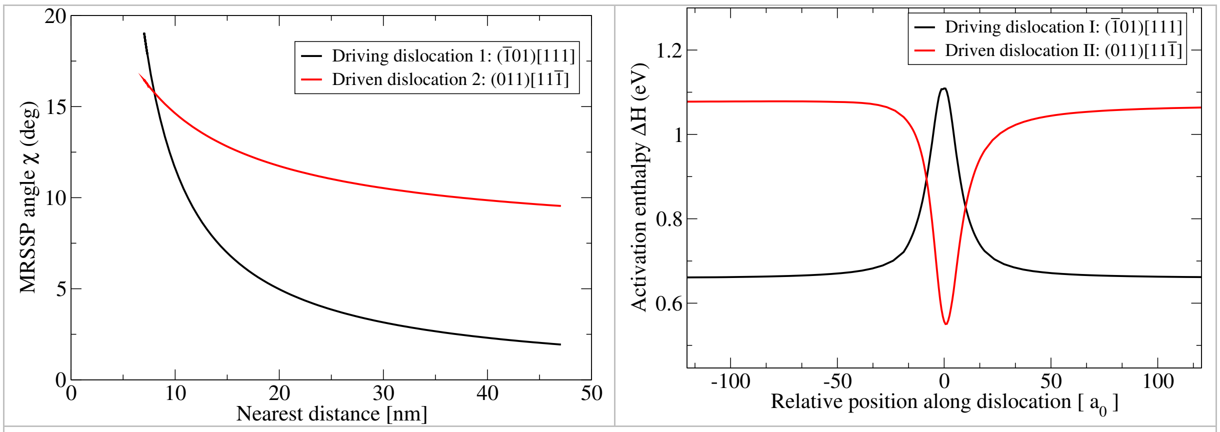

a) b)

Supplementary Figure 3: Influence of repulsive interaction on dislocation mobility parameters(a) MRSS angle vs. nearest distance for the $[111]-[11\bar{1}]$ dislocation system. Activation enthalpy plotted along the length of the dislocations I and II for the a/2[111]-$a/2[11\bar{1}]$ interacting screw dislocations. b) The activation enthalpy is computed for a nearest distance of about 7.3nm at which collective motion occurs. The zero-point corresponds to the point of nearest approach on the corresponding dislocations. The relative position is shown in units of lattice constant a_0.

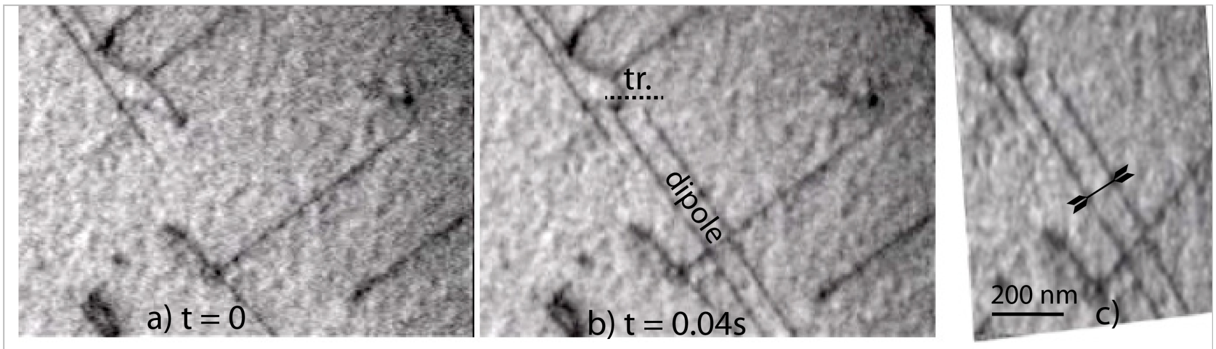

Supplementary Figure 4: Expanding screw dipole in tungsten strained at 300K. a) initial state, b) after expansion the screw dipole has emerged along the slip trace tr, c) same as (b) after correction from perspective effects: the dipole is seen in its plane and its critical width can be measured.

Supplementary Note 1 Interaction between a single screw and array of repulsive screw dislocations

The arguments presented in the main paper apply also to situations where one single screw dislocation interacts with many forest screw dislocations in a repulsive configuration: To test this idea a setup consisting of a primary screw dislocation on the $(\bar{1}01)[111]$ slip system (magenta) gliding through eight forest dislocations on the $(011)[11\bar{1}]$ (red) slip system as shown in Supplementary Fig. 5 is studied. The $(011)[11\bar{1}]$ slip system has a very low Schmid factor and cannot be expected to be activated by macroscopic stress-state along. In Supplementary Fig. 5 the configurations at different time steps are shown. The single screw dislocation successively collects and drives all eight forest dislocations in their respective glide planes at the same applied stress in a manner similar to the two-dislocation system. This shows that the local interaction effect plays a dominant role in the dislocation dynamics of screw dislocations in the temperature regime when the lattice friction is dominant. Hence in this regime, significant plastic flow occurs due to secondary activation of a very low-stressed plane and the repulsive forest dislocations may rather surprisingly lead to a decrease in the hardening rate.

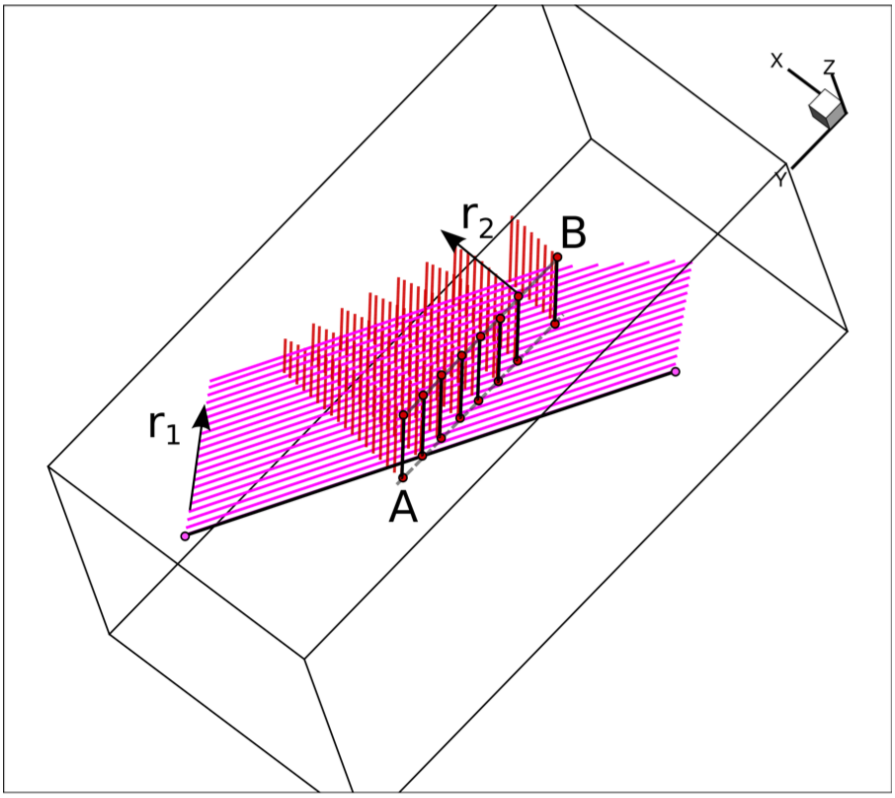

Supplementary Figure 5: **Time series of a dislocation pushing 8 forest dislocation**: An overlay of the coupled motion of a screw dislocation (magenta) on the $(\bar{1}01)[111]$ system with dislocations on the forest $(011)[11\bar{1}]$ (red) slip system is shown. The forest consists of eight repulsively oriented screw dislocations. Due to the applied stress state the glide direction of dislocation I and forest dislocations (dislocation II) is along direction $\mathbf{r}_1$ and $\mathbf{r}_2$ respectively. The initial positions of the primary (dislocation I) and forest screw dislocations (dislocation(s) of type II) are indicated with solid black lines between magenta and red points respectively. Filled magenta and red circles indicate the end points of the driving screw dislocation I with Burgers vector $[111]$ and the forest screw dislocations respectively in their initial positions. Grey lines A and B connect the end points of the forest screw dislocations with Burgers vector $[11\bar{1}]$ in their initial positions. The forest dislocations end at the surface of the sample.

Supplementary Note 2 Calculation of dislocation pairs

Slip system notation:

Only slip systems of type $\{110\}\langle 111\rangle$ are considered, based on atomistic results on possible slip systems [1] as shown in Supplementary Table 1. In the DDD mobility law evaluation scheme, the slip system notation used in Gröger 2008 [1] is employed, where only positive shear stresses occur. Therefore 24 instead 12 slip system are used, allowing to represent both positive and negative slip.

#no slip system	Burgers vector $\mathbf{b}$ $[.5a_0]$	Glide plane normal $\mathbf{n}$ $[\frac{1}{\sqrt{2}}]$
1	111	$01\bar{1}$
2	111	$\bar{1}01$
3	111	$1\bar{1}0$
4	$\bar{1}11$	$\bar{1}0\bar{1}$
5	$\bar{1}11$	$0\bar{1}1$
6	$\bar{1}11$	110
7	$\bar{1}\bar{1}1$	$0\bar{1}\bar{1}$
8	$\bar{1}\bar{1}1$	101
9	$\bar{1}\bar{1}1$	$\bar{1}10$
10	$1\bar{1}1$	$10\bar{1}$
11	$1\bar{1}1$	011
12	$1\bar{1}1$	$\bar{1}\bar{1}0$

#no slip system	Burgers vector $\mathbf{b}$ $[.5a_0]$	Glide plane normal $\mathbf{n}$ $[\frac{1}{\sqrt{2}}]$
13	$\bar{1}\bar{1}\bar{1}$	$01\bar{1}$
14	$\bar{1}\bar{1}\bar{1}$	$\bar{1}01$
15	$\bar{1}\bar{1}\bar{1}$	$1\bar{1}0$
16	$1\bar{1}\bar{1}$	$\bar{1}0\bar{1}$
17	$1\bar{1}\bar{1}$	$0\bar{1}1$
18	$1\bar{1}\bar{1}$	110
19	$11\bar{1}$	$0\bar{1}\bar{1}$
20	$11\bar{1}$	101
21	$11\bar{1}$	$\bar{1}10$
22	$\bar{1}1\bar{1}$	$10\bar{1}$
23	$\bar{1}1\bar{1}$	011
24	$\bar{1}1\bar{1}$	$\bar{1}\bar{1}0$

Supplementary Table 1: slip systems notation from [1]. Only systems with positive resolved shear stress are active (at most 12). a_0 is the lattice constant.

Dislocation junctions / pairs:

Now, we determine the number of repulsive pairs and classify them according to their attractive counterpart (dislocation junctions) in 6 groups following the notation of Queyreau et. al. [2]. The relevant types are highlighted in bold.

0) Slip system self-interaction: not relevant for repulsive configuration
1) Dipolar (same normal vector, but different Burgers vector): not relevant for repulsive configuration.
2) Collinear: leads to junction with zero Burgers vector. This requires, that one of the Burgers vectors is shared by both glide planes. This case is also not relevant, as cross-slip occurs easily and long range cooperative motion is not possible.
3) **Mixed symmetrical junction**: intersection line of glide planes $\mathbf{t}_{\mathrm{inter}}$ along $\langle 111\rangle$ direction; Burgers vector of reaction is of $\langle 100\rangle$ type.
   This combination is of importance: in case of repulsive line orientation (case study of the main paper DDD example) the coupled pair can glide parallel to $\mathbf{t}_{\mathrm{inter}}$ over long

distances. The vector $\mathbf{t}_{\text{short}}$ pointing between the location of nearest approach is parallel to a $\langle 110 \rangle$ direction. The intersection line $\mathbf{t}_{\text{inter}}$ and $\mathbf{t}_{\text{short}}$ are inclined.

4) Mixed asymmetrical junction: reaction Burgers vector is in one of the initial glide planes (reaction is of 100 type); this (as in case 2) means that one of the Burgers vectors is shared by both glide planes and thus does not allow for coupled motion over long distances.
5) **Edge junction:** This dislocation pair form edge dislocation junction (Burgers vector $\langle 010 \rangle$; the glide planes intersect along $\mathbf{t}_{\text{inter}} = \langle 100 \rangle$ directions; the reaction is sessile. In case of repulsive orientation, the vector $\mathbf{t}_{\text{short}}$ pointing between the location of nearest approach is parallel to a $\langle 101 \rangle$ direction. Also in this case a coupled motion over long distances is possible. This case corresponds to the experimental observation.

<u>Total count of possible forest pairs between 12 slip systems:</u>
(12*12-(no_of_diagonal) 12)/2-no_type_planar(6) = 60 pairs
The same holds for the 24 slip system notation.

Using 12 slip systems notation for the possible reactions, the following table is obtained
First row: slip system number
First column: slip system number:

	**1**	**2**	**3**	**4**	**5**	**6**	**7**	**8**	**9**	**10**	**11**	**12**
1:	0	2	2	4	1	4	5	3	4	4	5	3
2:	2	0	2	5	4	3	3	5	4	1	4	4
3:	2	2	0	3	4	5	4	4	1	4	3	5
4:	4	5	3	0	2	2	4	1	4	5	3	4
5:	1	4	4	2	0	2	5	4	3	3	5	4
6:	4	3	5	2	2	0	3	4	5	4	4	1
7:	5	3	4	4	5	3	0	2	2	4	1	4
8:	3	5	4	1	4	4	2	0	2	5	4	3
9:	4	4	1	4	3	5	2	2	0	3	4	5
10:	4	1	4	5	3	4	4	5	3	0	2	2
11:	5	4	3	3	5	4	1	4	4	2	0	2
12:	3	4	5	4	4	1	4	3	5	2	2	0

Counting within the yellow submatrix: number of different reactions

Type of interaction	no of occurrence	comments
0	12	Planar: not relevant here
1	6	Planar: not relevant here
2	12	not relevant here
**3**	**12**	**intersection $\mathbf{t}_{\text{inter}}$: along $\langle 111 \rangle$**<br>**$\mathbf{t}_{\text{short}}$: along $\langle 110 \rangle$**
4	24	not relevant here
**5**	**12**	**intersection $\mathbf{t}_{\text{inter}}$: along $\langle 100 \rangle$;**<br>**$\mathbf{t}_{\text{short}}$: along $\langle 110 \rangle$**

Repulsive dislocation configuration:

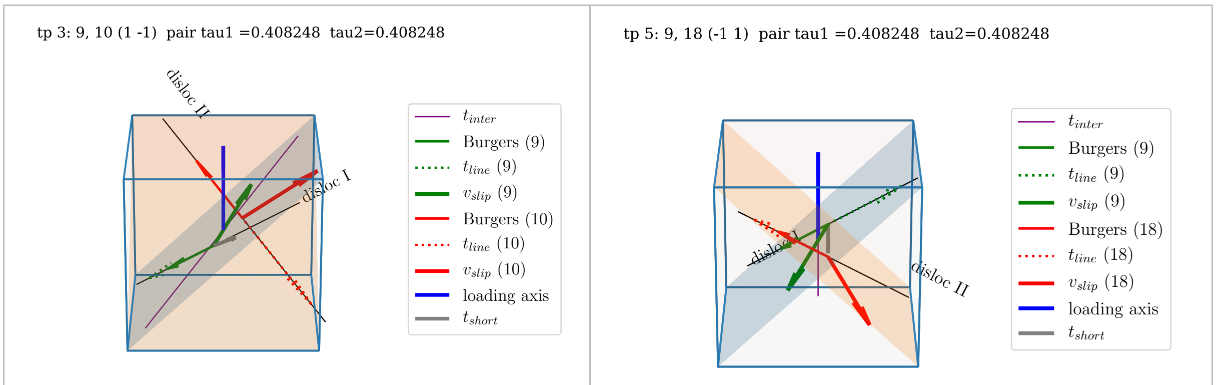

a) **b)**

Supplementary Figure 6: Repulsive pair types: example for a) type 3 repulsive configuration involving slip systems 9 and 10 and b) type 5 repulsive configuration involving slip systems 9 and 18. The resolved shear stresses [arb. units] correspond to an uniaxial loading along [100] direction (blue line).
(top line in a) tp 3: Type of interaction 3; 9,10: glide system number; (1 -1) sign of line direction $\mathbf{t}_{\text{line}}$ of dislocation with respect to Burgers vector direction); tau1 resp. tau2: resolved shear stress on slip system 9 resp. 10 slip system (in a)) of pair (here identical Schmid factor). Burgers (#no of slip system), $\mathbf{t}_{\text{line}}$(#no of slip system), vector $\mathbf{v}_{\text{slip}}$ (#no slip system) indicates the Burgers vector, line direction and glide direction of the screw dislocation in slip system (#no).

In Supplementary Fig. 6 the two types of repulsive configurations are shown for an uniaxial loading along the [100] direction of the crystal.

The pair-wise repulsive interaction of screw dislocation has consequences on the effective plastic slip. The observation by the DDD modelling shows that a screw dislocation may trigger the motion of another screw dislocation, which is (i) repulsively oriented with respect to the driving one and (ii) is loaded such that a coupled motion is possible.

Determination of repulsively oriented pairs:

To establish a list of active/relevant pairs depending on loading direction, the possible glide directions of both screw dislocations in each pair are characterized. The following additional conditions for a repulsive screw dislocation pair must be fulfilled in order to allow for a coupled motion under a given stress state:
The slip system (i) is defined by a Burgers vector $\vec{b}^{(i)}$ and a normal vector $\vec{n}^{(i)}$ using the following notation $(\vec{n}^{(i)}, \vec{b}^{(i)})$. The normalized Burgers vector is denoted by $\hat{b}^{(i)}$ and b is its norm.

Their mutual interaction is repulsive, e.g. for each slip system $(\vec{n}^{(i)}, \vec{b}^{(i)})$ one has to consider both line directions for the screw dislocations:

1. Pick system 1 $(\vec{n}^{(1)}, \vec{b}^{(1)})$ and line direction $\vec{t}^{(1)}$: search system 2 $(\vec{n}^{(2)}, \vec{b}^{(2)})$ with repulsive line direction $\vec{t}^{(2)}$
2. Calculate the slip direction $\vec{s}_{\mathrm{PK}}^{(i)}$ in their respective glide planes: the slip direction is given by $\vec{s}_{\mathrm{PK}}^{(i)} = \vec{n}^{(i)} \times \vec{t}^{(i)}$
3. If the scalar product $\vec{s}_{\mathrm{PK}}^{(1)} . \vec{s}_{\mathrm{PK}}^{(2)} > 0$ the dislocation would move into "similar directions" for non-zero glide stresses: cooperative motion is possible. The $\vec{s}_{\mathrm{PK}}^{(i)}$ is labeled "v_{slip} (#no of slip system)" in the Supplementary Fig. 6.

This leads to 24 possible repulsive pairs out of the total 60 irrespective of the loading. Depending on the stress state, the pairs are classified according to their resolved shear stresses/Schmid factor. Therefore we first calculate the resolved shear stresses:

- $\tau^{(i)} = \vec{n}^{(i)} \boldsymbol{\sigma} \hat{b}^{(i)}$
- the resolved Peach Koehler force points along direction $\vec{s}_{\mathrm{PK}}^{(i)}$:

$$\vec{F}_{\mathrm{PK}}^{\mathrm{resolved}} = \tau^{(i)} b \; \vec{s}_{\mathrm{PK}}^{(i)}$$

Classify the pairs and the most important ones are given first:

a. The resolved shear stress on both is larger zero: (type: both)
b. Only one of the resolved shear stresses is larger zero (type: one)
c. None of the resolved shear stresses are larger zero (type: none)

Primary slip plane

An additional constraint on the pairs has to be considered: only pairs where both dislocations are one their resp. primary glide plane can contribute to cooperative glide. The screw dislocation would cross-slip otherwise and destroy the pair.
Including this condition the number of pairs reduces further as detailed in the last column of Supplementary Table 2.

Overview on pair counting for different crystal orientations:

**Crystal orientation**	**Total number of repulsive pairs with product** $\vec{s}_{\mathrm{PK}}^{(1)}.\vec{s}_{\mathrm{PK}}^{(2)} > 0$	**Both:** $\tau_1 > 0$ **and** $\tau_2 > 0$	**One:** $\tau_1 > 0$ **or** $\tau_2 > 0$	**None**	**Primary systems only + finite Schmid factor on pushed system**
$\langle 100 \rangle$	24	12	8	4	#no total (both on primary): 12<br>*Type3: 12 (4 on primary)<br>*Type5: 8 (all 8 on primary)
$\langle 110 \rangle$	24	4	8	0	#no total (both on primary): 4<br>*Type3: 2 (all 2 on primary)<br>*Type5: 2 (all 2 on primary)
$\langle 111 \rangle$	24	3	18	0	#no total (both in primary): 3<br>Type3: 9 (3 on primary and both non-zero Schmid; 6 where one of the dislocations has zero Schmid factor)<br>Type5: 12 (one of the dislocations has zero Schmid factor)
$\langle 149 \rangle$	24	24	0	0	#no total (both in primary): 3<br>Type3: 12 (2 on primary)<br>Type5: 12 (1 on primary)

Supplementary Table 2: *Counting of pairs depending on loading direction. (or is exclusive or => xor).*

The Supplementary Table 2 shows that for high symmetry orientations the number of pairs where both dislocations have a non-zero Schmid factor and are in their resp. primary glide plane depends strongly on the loading direction.

Coupling factor between system I and II:

The direction cosine between the slip direction of the screw dislocation is used as a weighting factor of the coupling of both systems:

Type 3: $K_{\alpha\beta} = \vec{s}_{\mathrm{PK}}^{(\alpha)}.\vec{s}_{\mathrm{PK}}^{(\beta)} = 0.8\overline{3}$

Type 5: $K_{\alpha\beta} = \vec{s}_{\mathrm{PK}}^{(\alpha)}.\vec{s}_{\mathrm{PK}}^{(\beta)} = 0.6\overline{6}$

Detail on pairs depending on orientation

These tables list the pairs for uniaxial tensile loading in specific directions. Each lines describes the details of both dislocations of the pair: slip system information sys1, sys2; line direction with respect to Burgers vector direction of slip system (tb1,tb2); type of arrangement; Schmid factor/resolved shear stress (tau1,tau2) and a flag indicating of the screw dislocation is on its primary glide plane.

100 pairs:

no	sys1	sys2	tb1	tb2	type	tau1	tau2	primary1	primary2
**1**	1	20	1	-1	3	0	0.408	F	T
**2**	1	24	1	1	3	0	0.408	F	T
**3**	3	4	1	-1	3	0.408	0.408	T	T
**4**	3	11	1	-1	3	0.408	0	T	F
**5**	4	11	1	1	3	0.408	0	T	F
**6**	5	9	1	-1	3	0	0.408	F	T
**7**	5	10	1	1	3	0	0.408	F	T
**8**	7	14	1	-1	3	0	0.408	F	T
**9**	7	18	1	1	3	0	0.408	F	T
**10**	9	10	1	-1	3	0.408	0.408	T	T
**11**	14	18	1	-1	3	0.408	0.408	T	T
**12**	20	24	1	-1	3	0.408	0.408	T	T
**13**	3	18	-1	1	5	0.408	0.408	T	T
**14**	3	24	-1	1	5	0.408	0.408	T	T
**15**	4	10	-1	1	5	0.408	0.408	T	T
**16**	4	14	-1	1	5	0.408	0.408	T	T
**17**	9	18	-1	1	5	0.408	0.408	T	T
**18**	9	24	-1	1	5	0.408	0.408	T	T
**19**	10	20	-1	1	5	0.408	0.408	T	T
**20**	14	20	-1	1	5	0.408	0.408	T	T

Supplementary Table 3: 100 loading direction: 20 pairs (sys1,sys2) are the slip system according to Supplementary Table 1; tb1 resp. tb2 are the sign of the relative line directions of dislocation 1 resp. 2 of the pair with respect to its Burgers vector. Type indicates the repulsive configuration type (either 3 or 5). Tau1 resp. tau2 are the resolved shear stresses. Primary1 resp. primary2 indicates if screw dislocation is in its primary glide plane.

110 pairs:

no	sys1	sys2	tb1	tb2	type	tau1	tau2	primary1	primary2
**1**	1	20	1	-1	3	0.408	0.408	T	T
**2**	1	24	1	1	3	0.408	0	T	T
**3**	7	14	1	-1	3	0.408	0.408	T	T
**4**	7	18	1	1	3	0.408	0	T	T
**5**	14	18	1	-1	3	0.408	0	T	T
**6**	20	24	1	-1	3	0.408	0	T	T
**7**	1	7	-1	1	5	0.408	0.408	T	T
**8**	1	11	-1	-1	5	0.408	0	T	T
**9**	4	14	-1	1	5	0	0.408	T	T
**10**	5	7	-1	-1	5	0	0.408	T	T
**11**	10	20	-1	1	5	0	0.408	T	T
**12**	14	20	-1	1	5	0.408	0.408	T	T

Supplementary Table 4: 111 loading direction: 12 pairs ; details see Supplementary Table 3.

111 pairs:

no	sys1	sys2	tb1	tb2	type	tau1	tau2	primary1	primary2
**1**	2	6	1	-1	3	0	0.272	T	T
**2**	2	7	1	1	3	0	0.272	T	T
**3**	3	11	1	-1	3	0	0.272	T	T
**4**	3	16	1	1	3	0	0.272	T	T
**5**	6	7	1	-1	3	0.272	0.272	T	T
**6**	11	16	1	-1	3	0.272	0.272	T	T
**7**	13	20	1	1	3	0	0.272	T	T
**8**	13	24	1	-1	3	0	0.272	T	T
**9**	20	24	1	-1	3	0.272	0.272	T	T
**10**	2	16	-1	1	5	0	0.272	T	T
**11**	2	20	-1	-1	5	0	0.272	T	T
**12**	3	6	-1	-1	5	0	0.272	T	T
**13**	3	24	-1	1	5	0	0.272	T	T
**14**	5	7	-1	-1	5	0	0.272	F	T
**15**	5	11	-1	1	5	0	0.272	F	T
**16**	6	9	-1	-1	5	0.272	0	T	F
**17**	7	13	-1	-1	5	0.272	0	T	T
**18**	9	24	-1	1	5	0	0.272	F	T
**19**	10	16	-1	-1	5	0	0.272	F	T
**20**	10	20	-1	1	5	0	0.272	F	T
**21**	11	13	-1	1	5	0.272	0	T	T

Supplementary Table 5: 111 loading direction: 21 pairs ; details see Supplementary Table 3.

Remarks on the plastic strain caused by the driven dislocations:

With the above picture in mind, the additional plastic slip is solely due to free glide of the driven dislocation system and surprisingly no hardening effects are expected from this contribution. Therefore the additional plastic slip caused by collective motion is handled apart from the "standard" plastic slip contributions (see Supplementary Discussion "crystal plasticity model")

Role of Schmid factors: under high symmetry loading (both slip systems have the same Schmid factor / resolved shear stress), the role of pushing system resp. pushed system is not clearly defined. Due to the fact that each screw dislocation is differently embedded / connected (different screw dislocation length) the additional plastic slip due to repulsive interaction is expected to be of importance as local pinning sites/configuration can be overcome.

Cooperative effects:

The total number of coupled pairs depends on the loading direction. Now we want investigate if an avalanche like behavior may occur: is it possible that the pushed dislocation can now in turn push another one? This is done for the high symmetry orientations only, where both screw dislocations have the same non-zero Schmid factor but not the same embedding in the dislocation network.
In this case the trivial solution is that the pair (3,4) pushed the inverse pair (4,3) for a [100] loading direction.

The following types of chains or sequences of pushing pairs exist:

100 direction:

- Sequence with 3 or 4 pairs involved: the "end" slip system <u>differs</u> from the initial pushing dislocation
- with 4 pairs: the last pushed dislocation (slip system and line direction) is <u>identical</u> to the one of the initial screw dislocation

110 direction:

- sequence with 2 or 3 pairs involved: the end slip system <u>differs</u> from the initial pushing dislocation

111 direction:

- no sequence found

The 100 direction turned out to be the only one, where a closed sequence is found. This indicates that the cooperative effect might be most relevant for this orientation. In the Supplementary Discussion, the sequences are not taken into account.

Supplementary Discussion

Crystal Plasticity Model

The influence of the coupling presented in the main section is outlined here. The equations are solved by a standard 4th order Runge-Kutta scheme. This section serves to illustrate the possible consequences of the mechanism in a crystal plasticity model (CP).
In this section, we present briefly the crystal plasticity model and its adoptions to illustrate the effect of the coupled motion.

We start from the model by Bassani Wu (1991) [3] and just briefly outline the equations. The plastic slip rate on slip system α is $\dot{\gamma}^{\alpha}_{\mathrm{plast}}$. The subscript $(\quad)_{\mathrm{std}}$ is added for the quantities (plastic slip) used in the original (standard) crystal plasticity model. The slip systems are given in Supplementary Table 1.

The kinetic constitutive law for the shear rate $\dot{\gamma}^{\alpha}_{\mathrm{std}}$ of slip system α is [4] :

$$\dot{\gamma}^{\alpha}_{\mathrm{std}} = \dot{\gamma}_0 \left|\frac{\tau^{\alpha}}{\tau^{\alpha}_{\mathrm{c}}}\right|^{n} sign(\tau^{\alpha}) \tag{1}$$

where $\dot{\gamma}_0$ is a reference shear rate, $\tau^{\alpha}_{\mathrm{c}}$ the current strength (critical resolved shear stress) of slip system α and n is a materials parameter related to the strain rate sensitivity.

The evolution of the $\tau^{\alpha}_{\mathrm{c}}$ is described by

$$\dot{\tau}^{\alpha}_{\mathrm{c}} = \sum_{\beta} h_{\alpha\beta} \left|\dot{\gamma}^{\beta}\right| \tag{2}$$

Evolution equation for $\boldsymbol{\tau^{\alpha}_{\mathrm{c}}}$:

Model of Bassani Wu (1991): the change in the hardening behavior with plastic slip on the slip systems is:

$$h_{\alpha\alpha} = \left\{(h_o - h_{\mathrm{s}})\,/\cosh^2\left[\frac{(h_o - h_{\mathrm{s}})\gamma^{\alpha}_{\mathrm{std}}}{\tau_{\mathrm{s}} - \tau_0}\right] + \mathrm{h_s}\right\} * G\left(\{\gamma^{\beta}_{\mathrm{std}}\}; \beta \neq \alpha\right) \tag{3}$$

Off diagonal terms: $h_{\beta\alpha} = q h_{\alpha\alpha}$

- h_{s}: hardening module during easy glide within stage I
- $G\left(\{\gamma_{\beta}\}; \beta \neq \alpha\right)$: interaction/cross hardening

$$G\left(\gamma^{\beta}_{\mathrm{std}}; \beta \neq \alpha\right) = 1 + \sum_{\beta\neq\alpha} f_{\alpha\beta} \tanh\left(\frac{\gamma^{\beta}_{\mathrm{std}}}{\gamma_0}\right) \tag{4}$$

γ_0: is the amount of slip after which peak strength is reached

The current strength on glide system (α) with $\Delta\gamma^{\beta}_{\mathrm{std}} = \Delta t * |\dot{\gamma}^{\beta}_{\mathrm{std}}|$ and Δt is the time step used for integration:

$$\tau^{(\alpha)}_{\mathrm{c}} = \tau_o + \sum_{\beta} h_{\alpha\beta}\ \Delta\gamma^{\beta}_{\mathrm{std}} \tag{5}$$

Matrix $h_{\alpha\beta}$: weighting of the slip system interaction strength adapted from Queyreau et al. [5]. In the original work the application was done for high temperature bcc metals. Here it is applied for low temperature deformation (kink pair regime) and τ_0 is the initial strength (critical stress) on slip systems. In the original formulation the hardening matrix $h_{\alpha\beta}$ is used for a Taylor-like hardening term, whereas here we use it in a slip based formulation.

The above calculated plastic strains are the “classical/standard” contributions and are therefore labelled as $\gamma_{\mathrm{std}}^{\alpha}$. In the CP hardening law, only the plastic slip $(\quad)_{\mathrm{std}}$ is included.

The additional contribution to the total plastic slip, due to the coupling of screw dislocation motion is labelled $\gamma_{\mathrm{coupl}}^{\alpha}$.
Total plastic slip $\gamma_{\mathrm{tot}}^{\alpha}$ on slip system α is then given by $\gamma_{\mathrm{tot}}^{\alpha} = \gamma_{\mathrm{std}}^{\alpha} + \gamma_{\mathrm{coupl}}^{\alpha}$.
The effect of the coupled glide summed in $\gamma_{\mathrm{coupl}}^{\alpha}$ is obtained by a coupling matrix $K_{\alpha\beta}$ which describes if system β (dislocation I) pushed system α (dislocation II). For each deformation increment the contribution $\Delta\gamma_{\mathrm{coupl}}^{\alpha}$ is given by

$$\Delta\gamma_{\mathrm{coupl}}^{\alpha} = f_{\mathrm{coupl}} \sum_{\mathrm{Cooperative}\ (\beta,\alpha)} K_{\alpha\beta} \Delta\gamma_{\mathrm{std}}^{\beta} \qquad (6)$$

where the summation is done over cooperative pairs (β, α) of slip systems α (Supplementary Note 1). This involves the stress state, identifying pushing and pushed systems and type of coupling. The prefactor f_{coupl} allows to scale the effect and is set to 0.5 in the numerical examples shown below. Physically a value of 1 would mean, that each driving dislocation triggers a “repulsive” event. For the chosen value of 0.5 every other dislocation triggers such an event. The coupling factor directly scales the additional plastic slip contribution. As this additional contribution to the plastic slip does not lead to work-hardening, the flow stress is always lower compared to the one of standard CP for the same total strain.
For the numerical evaluation, we consider direct pairs only and not “chains or sequences” of pairs, whose occurrence and number are orientation dependent (Supplementary Note 1)
The total accumulated plastic slip due to cooperative motion is

$$\gamma_{\mathrm{coupl}}^{\alpha} = \sum_i \Delta\gamma_{\mathrm{coupl}}^{\alpha}|_i \qquad (7)$$

where i is the $\mathrm{i^{th}}$ integration step.

Non-Schmid Terms:

The expression $\tau_{\mathrm{eff}}^{\alpha}$ is used instead of the $\tau^{\alpha} = \boldsymbol{m}^{\alpha}\boldsymbol{\sigma}\boldsymbol{n}^{\alpha}$ alone to include the non-Schmid effects [6]: this results required to get the simulated curves closer to the experimental ones; especially the [111] deformation behavior.

$$\tau_{\mathrm{eff}}^{\alpha} = \boldsymbol{m}^{\alpha}\boldsymbol{\sigma}\boldsymbol{n}^{\alpha} + a_1\, \boldsymbol{m}^{\alpha}\boldsymbol{\sigma}\boldsymbol{n}_1^{\alpha} + a_2(\boldsymbol{n}^{\alpha} \times \boldsymbol{m}^{\alpha})\boldsymbol{\sigma}\, \boldsymbol{n}^{\alpha} + a_3\, (\boldsymbol{n}_1^{\alpha} \times \boldsymbol{m}^{\alpha})\boldsymbol{\sigma}\, \boldsymbol{n}_1^{\alpha} \qquad (8)$$

material	a_1	a_2	a_3
**W**	0	0.56	0.75

Parameter identification:

The standard model without the coupling term has been adapted to pick up the early initial yielding for [100] loading and the maximal flow stress for [111] orientation, which required the inclusion of non-Schmid effects. The identified parameters are

$\dot{\gamma}_0 = 10^{-4}/s$; n=20;

$\tau_c^\alpha = 125\text{MPa}$ is chosen as initial value as yielding begins around 300MPa for the [100] orientation.

Hardening coefficient $h_0 = 7500\ MPa$

Q=0.025: some latent hardening

$\gamma_0 = 0.001$: rather small value as no transition from easy glide is observed: stage II

$\tau_s = 155\text{MPa}$: staturation stress for stage I

$h_s = 100\ \text{MPa}$

Interaction coefficients $f_{\alpha\beta} = f_0 f_{\text{type}(\alpha,\beta)}$ where $\text{type}(\alpha, \beta)$ stand for one of the reaction types in Supplementary Notes 1 and 2.

f_0	Type 0	Type 1	Type 2	Type 3	Type 4	Type 5
0.06	2.5	2.5	65	6.5	20	6.5

The relative weight of the $f_{\text{type}(\alpha,\beta)}$ follows the table in [5]. The prefactor is adapted to the experimental curves.

Observation from Supplementary Fig. 7

- ⇨ The onset of plasticity is rather early for [100] in both experiments and simulation
  - Switching on the coupling term leads to an extended work hardening regime CP(coupl) which is in rather good agreement with the experimental curve
- ⇨ For [111]: the initial hardening in the CP model is not changed significantly
  - The peak values are close to the experimental ones
  - Inclusion of non-Schmid are especially relevant for this orientation; with Schmid only the flow stress would be ~30% larger. (not included in figure)
- ⇨ Also the [110] hardening slope is changed: goes further away from the experiments:
  - This orientation is rather particular for several reasons: little to no dislocation density increase is observed in experiments [7] and the sudden transition from elastic to almost ideal plastic may be due to the lack of mobile dislocations. Furthermore, according the experimental observations one should also consider slip on {112} planes [8,9] for [110] loading. The current analysis does not include those systems.

The saturation level is similar; the hardening rate for larger strains is reduced and therefore closer to the experimental values, where no hardening is observed.

Stress strain curve from CP model:

[100] with experiment

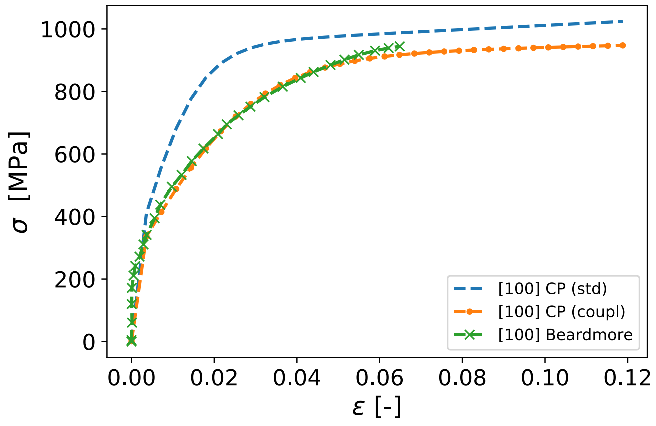

[111] with experiments

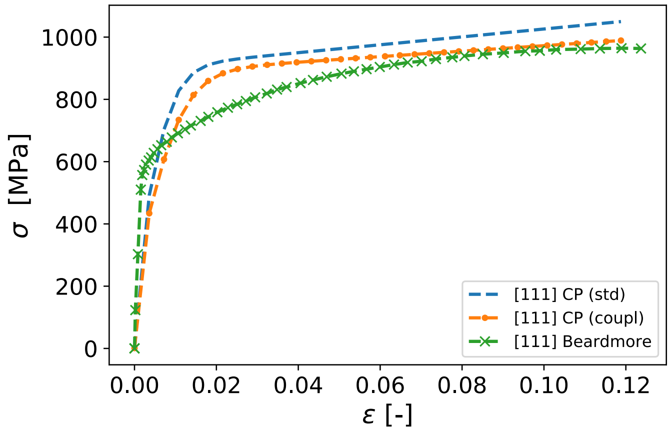

[110] with experiment

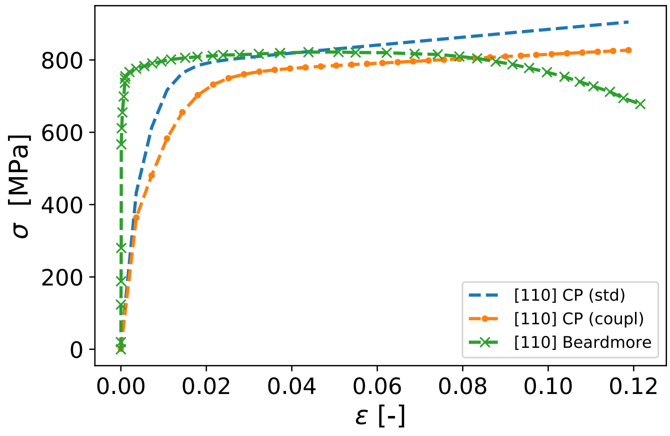

Supplementary Figure 7: stress strain curve : (no) means no coupling term; (with) with coupling term switched on; (f_{coup} = 0.5: slip on pushing generates half the slip on pushed system)

Simulation results from an atomistically-informed crystal plasticity model of Cereceda et al.[10] for tungsten show for all orientations considered above a sudden change from the elastic to plastic behavior, thus leaving the origin of the extended work hardening regime open. The coupling mechanism proposed here allows to explain this phenomenon.

Supplementary References

1. Gröger, R., Bailey, A. G. & Vitek, V. Multiscale modeling of plastic deformation of molybdenum and tungsten: I. Atomistic studies of the core structure and glide of 1/2 〈111〉 screw dislocations at 0K. *Acta Mater.* **56**, 5401–5411 (2008).
2. Queyreau, S., Monnet, G. & Devincre, B. Slip systems interactions in α-iron determined by dislocation dynamics simulations. *Int. J. Plast.* **25**, 361–377 (2009).
3. Bassani, J. L. & Wu, T.-Y. Latent Hardening in Single Crystals II. Analytical Characterization and Predictions. *Proc. R. Soc. A Math. Phys. Eng. Sci.* **435**, 21–41 (1991).
4. Rice, J. R. R. Inelastic constitutive relations for solids: An internal-variable theory and its application to metal plasticity. *J. Mech. Phys. Solids* **19**, 433–455 (1971).
5. Queyreau, S., Monnet, G. & Devincre, B. Slip systems interactions in α-iron determined by dislocation dynamics simulations. *Int. J. Plast.* **25**, 361–377 (2009).
6. Gröger, R., Racherla, V., Bassani, J. L. & Vitek, V. Multiscale modeling of plastic deformation of molybdenum and tungsten: II. Yield criterion for single crystals based on atomistic studies of glide of 1/2〈111〉 screw dislocations. *Acta Mater.* **56**, 5412–5425 (2008).
7. Beardmore, P. & Hull, D. Deformation and fracture of tungsten single crystals. *J. Less-Common Met.* **9**, 168–180 (1965).
8. Argon, A. . & Maloof, S. . Plastic deformation of tungsten single crystals at low temperatures. *Acta Metall.* **14**, 1449–1462 (1966).
9. Caillard, D. Geometry and kinetics of glide of screw dislocations in tungsten between 95K and 573K. *Acta Mater.* **161**, 21–34 (2018).
10. Cereceda, D. *et al.* Unraveling the temperature dependence of the yield strength in single-crystal tungsten using atomistically-informed crystal plasticity calculations. *Int. J. Plast.* **78**, 242–265 (2016).
