# Supplementary material for: Repulsion leads to coupled dislocation motion and extended work hardening in bcc metals
Source: Nat Commun. 2020 Oct 9;11:5098. doi: 10.1038/s41467-020-18774-1 (PMC7547675; doi:10.1038/s41467-020-18774-1)
Supplement: Supplementary file 3 — Description of Additional Supplementary Files [file 41467_2020_18774_MOESM3_ESM.pdf]

Description for Additional Supplementary Files

Title: Supplementary Movie 1

Description: The movie shows the TEM observations of the dislocation motion in tungsten of Figure 3.
